# Screening, Diagnosis and Early Intervention in Autism Spectrum Disorders

**DOI:** 10.3390/children9020153

**Published:** 2022-01-25

**Authors:** Yurena Alonso-Esteban, Francisco Alcantud-Marín

**Affiliations:** Department of Developmental and Educational Psychology, University of Valencia, 46010 Valencia, Spain; Yurena.Alonso@uv.es

The increment of prevalence is among the most important changes that have taken place in recent years with regard to Autism Spectrum Disorder (ASD); in the 1970s and 1980s of the 20th century, the prevalence of ASD was estimated to be 4/10,000 [1], whereas nowadays, it is estimated to be between 1 and 2% [2,3]. In the 1970s and well into the 1980s, for most individuals with autism, it was considered that they needed lifelong residential or institutional care due to the insufficient improvements that were offered by the treatments of that time. Less than 2% of individuals with autism had a chance of living a normal life [1]. In the 1990s, ASD was still defined as “a severe and chronic developmental disorder, which with the current knowledge has no cure, its symptoms manifest themselves in different ways in different ages, accompanying the person during the whole life cycle” ([4] p. 37). Nowadays, Autism Spectrum Disorder (ASD) is considered to be one of the most common neurodevelopmental disorders. However, many gaps in the knowledge remain, particularly in relation to the etiology and pathogenesis of the disorder. There is irrefutable evidence that it is a biological and genetically based disorder [5]. The most promising results have undoubtedly been shown in the development of educational treatments [5,6]. One particular piece of evidence regarding the results is that currently, approximately 1% of people diagnosed with ASD before the start of schooling reach higher education [7].

To date, there is no biological marker of the disorder, so both detection and diagnosis are developed based on the observation of the symptoms described in the diagnostic manuals in use; in our case, detection and diagnosis are based on the DSM 5 [8] or ICD 11 [9]. According to both criteria, the symptoms of ASD must be present before the age of three. The early detection of ASD allows intervention to be initiated even before a formal diagnosis is made, at a critical time in neurodevelopment, which consequently causes better outcomes and prognosis [10,11,12,13]. For early intervention to be possible, early detection is necessary [14].

Screening consists of a procedure by which we separate healthy cases from those who present symptoms or suspicions of some type of disorder or disease. Screening is usually a quick and inexpensive procedure used to identify whether a patient has any of the signs described as symptoms of ASD. In 1999, the Child Neurology Society and the American Academy of Neurology already proposed to formulate “Practical Parameters for the Diagnosis and Evaluation of Autism”, with the coordination of the National Institute of Health of the United States and with the collaboration of several organizations of parents and professionals. More than 2500 relevant scientific articles were analyzed, concluding that the detection and correct diagnosis of ASD should be carried out at two levels: routine developmental surveillance and the diagnosis and assessment of ASD [15]. Screening instruments are usually classified into these two levels: those of universal application or level I instruments that only detect a “developmental delay” [16] and those of level II, which are usually specific to ASD or another disorder [17]. The current challenge is how the age of detection of ASD can be lowered to below 24 months without increasing false positives. Currently, there are at-risk groups such as siblings of already diagnosed children, children born preterm or children who are developmentally delayed. The follow-up of these risk groups in comparison to non-risk children allows us to better understand the pathogenesis of the disorder and to find new, earlier screening systems.

Although currently, the diagnosis of ASD could be stably established at around 20–24 months of age [18,19,20,21], there is often still a considerable delay between the time of the first suspicions detected by parents, the diagnosis and the start of intervention [22]. Although efforts have been made, up to 25% of autistic children are diagnosed at the beginning of their school years [23], with the average age of diagnosis being six years old [24]. The only reason for this delay is the lack of resources at the health, educational and social levels.

Now, there is an international consensus that the ADOS-2 (Autism Diagnostic Observation Schedule [25]) and ADI-R (Autistic Diagnosis Interview Revised [26]) are the gold standards for the diagnosis of ASD. This recognition does not mean that there are no other tools, such as the CARS (Childhood Autism Rating Scale [27,28]), GARS (Gilliam Autism Rating Scale [29]), DISCO (Diagnostic Interview for Social and Communication Disorders [30] or computerized tools such as 3DiA (Diagnostic Autism Rating Scale) [31].

Early intervention is understood as intervention carried out before the start of compulsory schooling, which, depending on the country and legislation, is usually established at around the age of six. There is ample evidence that demonstrates the influence of the age of onset on treatment, although there are also studies that suggest that the age of onset may have an influence on treatment [32,33,34]. A constant finding in studies on the effectiveness of early intervention programs is undoubtedly the lack of consistency of clinical trials due, among other reasons, to methodological reasons (small subject groups), the diversity of symptoms and severity levels of the disorder, different types of intervention and different intervention settings (clinic, school, home, etc.) and different measures of intervention outcomes, among others [35,36,37].

There is debate about which characteristics of a child and family may justify the success or best outcome of the intervention (age, language proficiency, IQ, family cohesion, resilience or adherence to treatment, etc.) [38,39]. However, it is well known that neuropsychological development is determined by interaction with the environment [40] and also that autistic children, as a consequence of an alteration in neurodevelopment, have deficits in the basic skills of communication and social interaction with their parents. Intervention at an early age would reduce the cascade effects [41]. Optimal effectiveness is found when we combine intervention with children with parental training programs [42,43].

The effectiveness of techniques and methods based on child development and the application of the principles of behavior analysis [44], including more evolved programs that emphasize the use of structured learning scenarios, stimulus control, the development of routines, natural environments, etc., is recognized [45]. The results suggest that children who receive these types of evolved programs achieve greater development of intelligence, social skills, communication and language and generally improve their quality of life [37,46,47,48,49] and consequently that of their families.

Research is directed towards enhancing the knowledge of the precursors of ASD symptoms with the aim of constructing a pathogenesis model on which to build more effective screening and diagnostic instruments; for example, we can cite the recent appearance of the ESB (Early Sociocognitive Battery [50]), which attempts to anticipate the age of diagnosis by focusing on the assessment of the socio-communicative development process down to two years. In the same vein, an extraordinary effort is being made to develop complementary assessment measures in an attempt to determine a cognitive profile that can be used both to aid diagnosis and to guide treatment.

Another line of research is related to gender. Due to a certain conviction that autism is a “male” condition, the presentation of autism in girls has been underestimated. However, in recent years, there has been great interest in finding an explanation as to why there are more diagnoses in boys than in girls. The lines of research are directed in two directions: toward the determination of a differential, symptomatological profile in the disorder and toward the genetic and biological explanation of these differences. In any case, a starting point is the consideration that diagnostic instruments may be biased toward girls [51].

In summary, the lines of research in detection and diagnosis are directed toward:

- The development of tools that contain precursors to the symptoms of ASD rather than the symptoms themselves in order to lower the age of detection.
- The development of new gender-sensitive screening and diagnostic tools or systems.
- The search for alternative or complementary detection systems to observation (biological, physiological, eyes-tracking, brain imaging, etc.), with the aim of improving sensitivity and bringing forward the age of diagnosis.
- The development of mathematical models to improve the sensitivity and specificity of the instruments.
- Increasing the accessibility to screening procedures using digital platforms.

Regarding early intervention programs, it is important to note that most of the evidence supporting the impact of early intervention comes from efficacy studies developed in university clinical trials. University clinical trials may use more resources than those available in community centers [52] or introduce biases related to the variability of cases or the attitudes and socioeconomic levels of families, so translational research is needed to bring the results closer to community intervention settings.
